# Effect of pulsed electromagnetic field therapy in patients undergoing total knee arthroplasty: a randomised controlled trial

**DOI:** 10.1007/s00264-013-2216-7

**Published:** 2013-12-20

**Authors:** Paolo Adravanti, Stefano Nicoletti, Stefania Setti, Aldo Ampollini, Laura de Girolamo

**Affiliations:** 1 Orthopaedic Surgery Department, Clinic “Città di Parma”, Parma, Italy; 2 IGEA Clinical Biophysics, Carpi, Italy; 3 Orthopaedic Biotechnology Laboratory, Galeazzi Orthopaedic Institute, Milan, Italy; 4 Orthopaedic Biotechnology Laboratory, IRCCS Istituto Ortopedico Galeazzi, Via R. Galeazzi 4, 20161 Milan, Italy

**Keywords:** Pulsed electromagnetic fields, Total knee replacement, Pain, Inflammation, Long-term result

## Abstract

**Purpose:**

It has been reported that even one year after total knee arthroplasty (TKA), a relevant percentage of patients does not attain complete recovery and indicate unfavourable long-term pain outcome. We compared the clinical outcome of 33 patients undergoing TKA randomly assigned to the control or the pulsed electromagnetic field group (I-ONE therapy).

**Methods:**

I-ONE therapy was administered postoperatively four hours per day for 60 days. Patients were assessed before surgery and then at one, two and six months postoperatively using international scores.

**Results:**

One month after TKA, pain, knee swelling and functional score were significantly better in the treated compared with the control group. Pain was still significantly lower in the treated group at the six month follow-up. Three years after surgery, severe pain and occasional walking limitations were reported in a significantly lower number of patients in the treated group.

**Conclusions:**

Advantages deriving from early control of joint inflammation may explain the maintenance of results at follow-up. I-ONE therapy should be considered an effective completion of the TKA procedure.

## Introduction

In recent decades innovations in total knee arthroplasty (TKA) biomaterials, design and surgical techniques have increased the rate of patient satisfaction. However, 11–25 % of patients report a noticeable improvement with respect to preoperatively but are still not satisfied with their TKA [1, 2]. It has been reported that even one year after surgery, a relevant percentage of patients (37 %) have not attained complete recovery [3] and indicates unfavourable long-term pain outcome (10 %–34 %) [4]. It has been suggested that early pain control and joint inflammation resolution following TKA may prevent long-term pain and functional limitations in uncomplicated TKAs [5]. Indeed, the strong local inflammatory component, which is present for some weeks after TKA surgery, may affect soft-tissue healing, leading to excess fibrous tissue, hypertrophic synovitis and heterotopic ossification that sustains chronic inflammation of the joint [6]. Pro-inflammatory mediators are directly responsible for several clinical symptoms that reflect the structural progression of osteoarthritis [7]. In particular, a significant correlation between interleukin-6 (IL-6) concentrations in joint fluid and functional scores was found one month after TKA, demonstrating that an intense inflammatory response is associated with a slow recovery [8]. It has also been demonstrated that high pre-operative synovial fluid concentrations of tumor necrosis factor-α (TNF-α), metalloproteinase-13 (MMP-13) and IL-6 in the knee are predictive of delayed pain resolution two years following TKA [9]. Moreover, a significant increase of IL-1β levels in the synovial fluid has been observed following TKA [10]. Therefore, the synovial membrane appears to be a promising target for novel strategies to control inflammation, prevent structural joint alterations and treat clinical symptoms, such as pain and swelling, after knee surgery [7]. Varani et al. [11], after having demonstrated the presence of A_2A_ or A_3_ adenosine receptors in human-fibroblast-like synoviocytes, showed that their modulation could be used to control inflammatory joint diseases [12]. Indeed, pulsed electromagnetic fields (PEMFs) show agonist activity for A_2A_ and A_3_ adenosine receptors, indicating that they can be used to control local inflammation and manage joint diseases [11, 12]. In human osteoarthritic fibroblasts, PEMFs increase the activation of these receptors reducing the synthesis of inflammatory mediators such as prostaglandin E_2_ (PGE_2_) and IL-6 and -8 [13].

Two prospective randomised double-blind trials demonstrated that patients using PEMFs after knee arthroscopy had a faster functional recovery and resolution of joint inflammation in comparison with patients in the placebo group [14, 15]. PEMF was also effective when used for two months after TKA, favoring functional recovery and early pain relief [16].

The primary objective of this randomised controlled trial in patients undergoing TKA stimulated with PEMF was to evaluate pain relief during follow-up; the secondary aim of the study was to determine whether PEMF could reduce joint swelling and accelerate functional recovery. A phone survey was performed to evaluate the number of patients still experiencing pain and functional limitation three years post-TKA.

## Materials and methods

### Patient characteristics

Patients with indication for TKA and satisfying the following inclusion criteria were enrolled: age between 60 and 85 years, grade 4 osteoarthrosis (Kellgren-Lawrence classification), varus/valgus malalignment <20° and 15°, respectively, and flexion deformity <15°. Previous surgery on the same knee, total hip arthroplasties, rheumatoid arthritis, autoimmune diseases, systemic diseases, tumors, severe malalignment and body mass index (BMI) > 30 kg/m^2^ were considered exclusion criteria. The study was approved by the Institutional Review Board and registered at Current Controlled Trials (www.controlled-trials.com; doi:10.1186/ISRCTN98459871). All the patients signed the informed consent.

### Randomisation process and I-ONE therapy protocol

Patients were randomly assigned to the treated or control group using a web-based randomisation program built on the randomisation criteria: sex (M/F), age (60–75 years; 75–85 years) and smoking status (yes/no). Patients allocated to the treated group by a research assistant not involved in patient assessment were instructed to use PEMF (I-ONE therapy, IGEA, Carpi, Italy) for four hours per day for 60 days. Treatment was started within seven days from surgery. The coils, placed on the operated knee, were powered by the PEMF generator system, which produces a pulsed signal with the following parameters: peak magnetic field intensity 1.5 ± 0.1 mT; frequency 75 Hz (patented). The patient could wear the battery-operated device during day or night and was instructed to interrupt treatment in case of adverse events, such as a burning sensation or skin irritation. The principal investigator and all physicians in charge of clinical controls were blinded to patient allocation.

### Surgical procedure and rehabilitation program

All patients received a cemented posterostabilised (PS) TKA (Vanguard®, Biomet, Warsaw, IN, USA) with patella resurfacing using a tourniquet and a medial parapatellar approach without patella eversion. All surgeries were performed by the same senior surgeon and the same anaesthesiologist. Patients were anaesthetised by selective spinal subarachnoid injection and positioning of an indwelling catheter at the femoral nerve level. In all patients, infiltration of the capsule with a periarticular injection of 120 ml ropivacaine 0.2 % 250 mg and epinephrine 1/1,000 0.5 mg was performed. To improve pain control and reduce the need for nonsteroidal anti-inflammatory drugs (NSAIDs) in the immediate postoperative period, elastomeric endovenous pump with 40–60 ml of ketoprofen and 20–30 ml of morphine and femoral perineural catheter with 0.3 % naropin for 48 hours were administered. Both patient groups followed a standard rehabilitation protocol: active and passive mobilisation of hip, knee and ankle using Kinetec (Rioveggio, Italy) from day one after surgery, accordingly to pain tolerance; from day two, assisted walking with two crutches; progressive weight-bearing regimen with discontinuation of crutch use according to each patient.

### Radiological assessment

To evaluate knee alignment, before surgery and a few days postsurgery, coronal-plane alignment was measured from standard weight-bearing anteroposterior (AP) radiographs with the knee in full extension [17].

### Clinical evaluation

All patients were clinically evaluated before surgery and then at one, two and six months after TKA. At each control, visual analogue scale (VAS) for pain, Knee Society Score (KSS) clinical rating system, the Short Form 36 (SF-36) Health Survey questionnaire were determined. Moreover, joint swelling was evaluated accordingly to Soderberg [18] using the following scores: 40, no difference between knee girth; 30, <0.5 cm; 20, between 0.5 and 1 cm; 10, between 1 and 1.5 cm; 0, >1.5 cm. An average of 3 years of follow-up phone surveys was performed: KSS function, persistence of pain and use of NSAIDs were recorded for each patient.

### Statistical analysis

Pain (VAS) was considered the primary outcome and used to calculate sample size of the study. According to the literature [14–16], a difference in mean VAS of 2 units between the two groups after the first month of therapy, with a standard deviation (SD) of 2 units, was hypothesised. To reach a statistical significance of 95 % with a power of 80 %, the sample size of each study group was equal to 15 subjects. Student’s *t *test was used to calculate mean variations for each parameter at each follow-up, with respect to baseline and to compare groups. Data are expressed as mean ± SD; *p* < 0.05 was considered statistically significant.

## Results

**Fig. 1:**
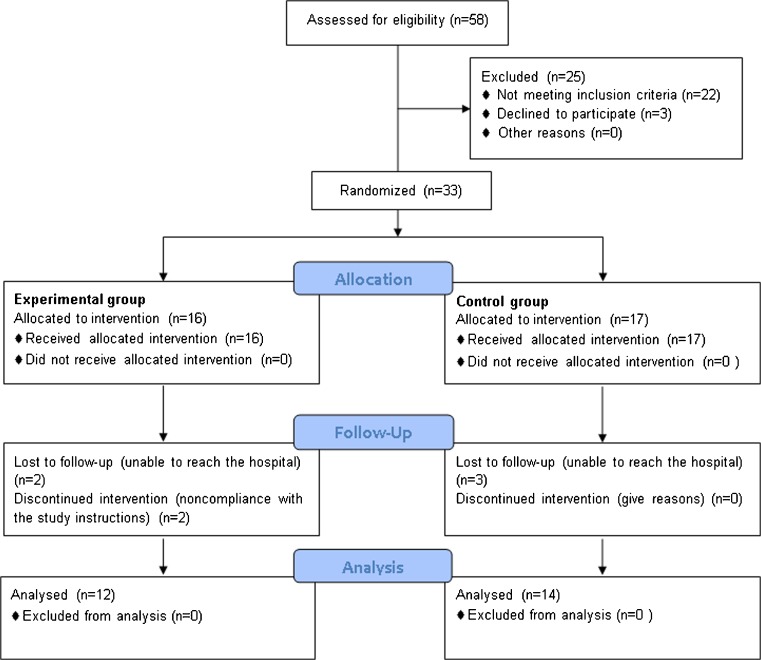
Consolidated Standards of Reporting Trials (CONSORT) flow chart for patient enrolment

Fifty-eight consecutive patients with indication for TKA were assessed for eligibility, of which only 33 (18 males, 15 females) matched the inclusion criteria. Data about 26 patients (12 treated group, 14 control group) were then available for final analyses (Fig. 1). At the time of enrollment the background population features were homogeneous between groups (Table 1).

**Table 1:** Pre-operative evaluation of study participants

	Treated group (*n* = 16)	Control group (*n* = 17)	*P* value
Sex	10 F, 6 M	9 F, 8 M	n.s.
Age (years)	66 ± 13	73 ± 5	n.s.
Height (cm)	169 ± 5	167 ± 10	n.s.
Weight (kg)	78 ± 11	77 ± 12	n.s.
KSS function score	51 ± 18	48 ± 11	n.s.
KSS knee score	12 ± 12	11 ± 20	n.s.
Knee swelling	24 ± 5	26 ± 6	n.s.
VAS	6.1 ± 2.0	5.8 ± 1.6	n.s.
SF-36	33 ± 12	37 ± 11	n.s.

*KSS* Knee Society Score, *VAS* visual analogue scale,* SF-36* Short-Form Health Survey questionnaire,* n.s*. not significant

I-ONE therapy was well tolerated by all patients, with no side effects.

### Coronal alignment

Varus and valgus knees were equally distributed in the two groups before surgery (not significant). Postoperative coronal alignment was 3.5° and 2.6° for treated and control group, respectively (not significant).

### VAS pain

**Fig. 2:**
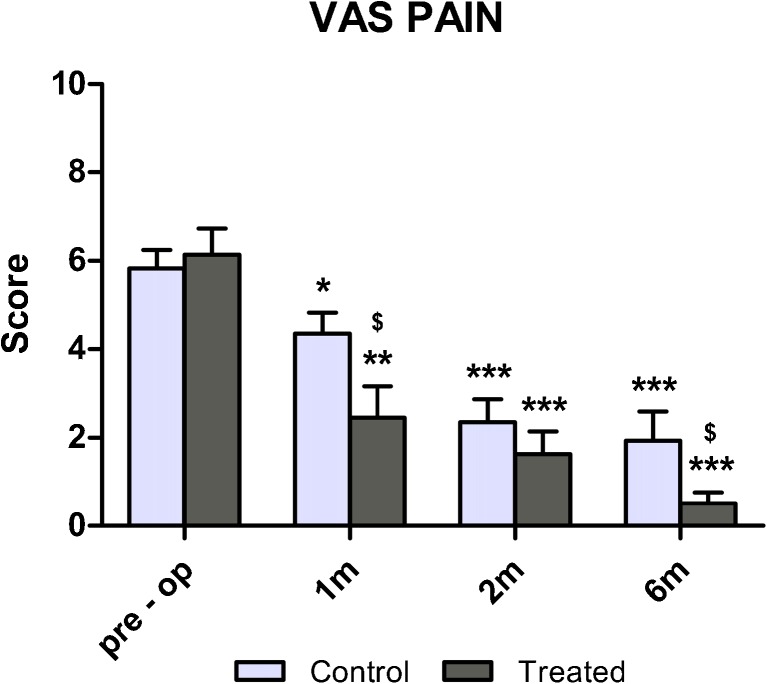
Visual analogue score (VAS) pain evaluation of patients before and 1, 2 and 6 months after total knee arthroplasty (TKA). **p < 0.01; ***p < 0.001 for each follow-up vs baseline; $*p* < 0.05 for the pulsed electromagnetic field (I-ONE) therapy group vs control group

Pain reduction was statistically significant for both groups with respect to preoperative level one month after TKA, although it was more evident in the treated group (−61 %, *p* < 0.001 and −26 % *p* < 0.05 for treated and control groups, respectively). Indeed, a significant difference between groups was observed at the one month follow-up in favour of the treated group (*p* < 0.05). Throughout the study, patients in the treated group showed lower VAS values in comparison with those in the control group, and at six months this difference was again significant (*p* < 0.05), with a 90 % pain reduction from baseline in the treated group. Moreover, it is noteworthy that the average VAS value of the treated group one month after surgery was comparable with that registered for the control group at six months (2.4 ± 2.3 and 1.9 ± 2.0, respectively; not significant) (Fig. 2).

### KSS: function and knee score

**Fig. 3:**
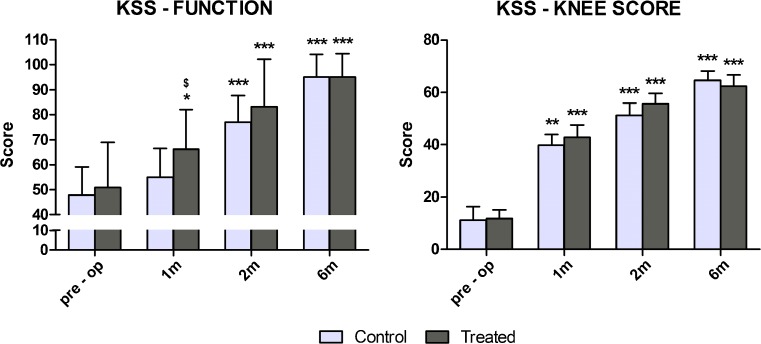
Knee Society Score (knee and function scores) of patients before surgery and at 1, 2 and 6 months after total knee arthroplasty (TKA). **p < 0.01; ***p < 0.001 for each follow up vs baseline. $*p* < 0.05 for the pulsed electromagnetic field ( I-ONE) therapy group vs control group

One month after surgery, the treated group showed a significant improvement in comparison with the preoperative value (+30 %; *p* < 0.05), whereas the control group presented comparable values with respect to baseline (+15 %; not significant). As a consequence, the difference between groups was statistically significant, with higher scores in the treated group (*p* < 0.05). Two and six months after surgery the functional score of both groups significantly improved with respect to baseline (*p* < 0.001), with no significant difference between groups (Fig. 3, left side). Also, the KSS knee score significantly improved in both groups beginning from one month after TKA (*p* < 0.001), with a constant increase over time but with no significant difference between groups (Fig. 3, right side).

### Swelling

**Fig. 4:**
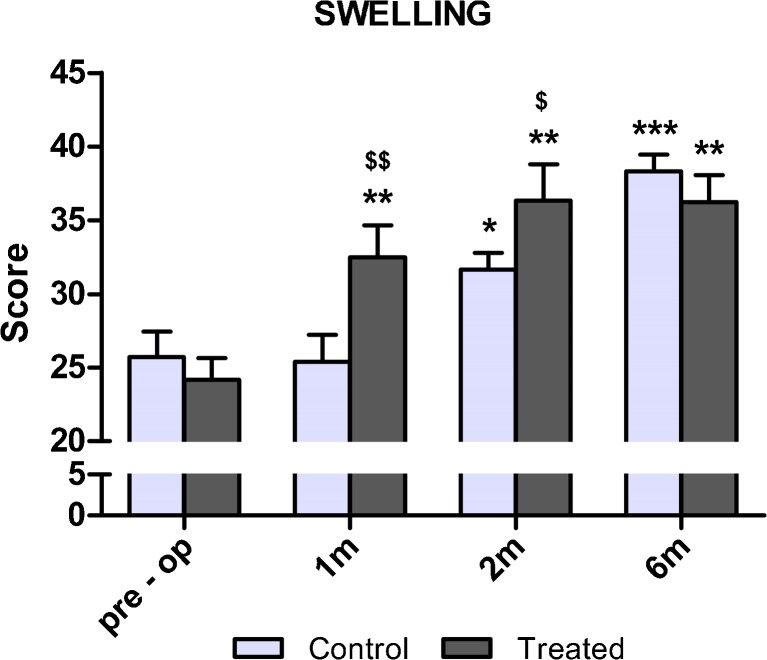
Swelling evaluation of patients before and 1, 2 and 6 months after total knee arthroplasty (TKA). Swelling, evaluated on palpation by the physician, is described using a scale ranging from 0 to 40, where higher scores indicate better results. *p < 0.05; **p < 0.01; ***p < 0.001 for each follow-up vs baseline. $*p* < 0.05; $$*p* < 0.01 for the pulsed magnetic field (I-ONE) therapy group vs control group

One month after TKA, swelling evaluation showed significantly better results in comparison with baseline values in the treated group (−34 %; *p* < 0.01). On the other hand, patients in the control group presented comparable values with respect to baseline. Indeed, significantly better results were observed in the treated group at the 1- (*p* < 0.01) and two month follow-up (*p* < 0.05). Knee swelling resolution was observed in both groups at six months (Fig. 4).

### SF-36 Health Survey

**Fig. 5:**
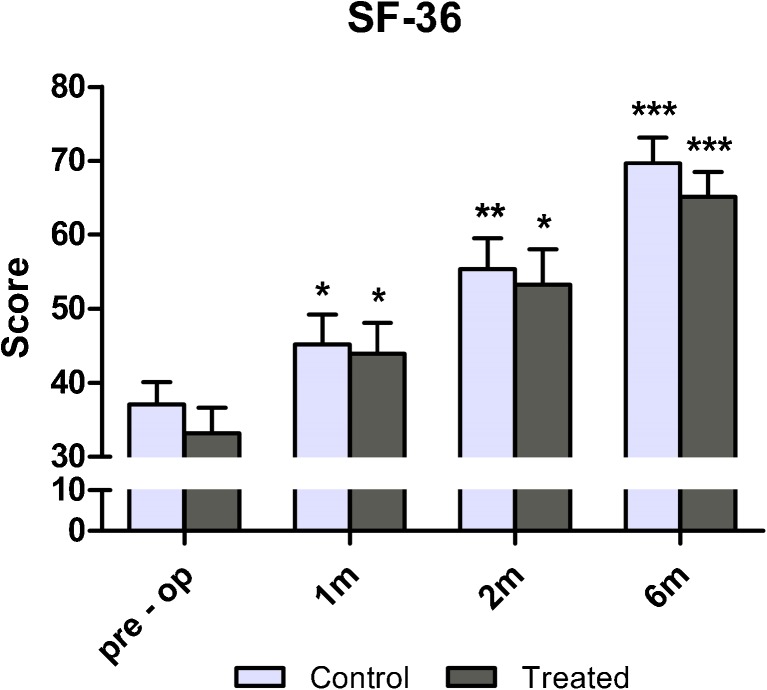
Short-Form Health Survey of 36 questions (SF-36) evaluation of patients before and 1, 2 and 6 months after total knee arthroplasty (TKA). *p < 0.05; **p < 0.01; ***p < 0.001 for each follow-up vs baseline

One month after surgery, the SF-36 of both groups significantly increased in comparison with baseline (+32 % and +22 % for treated and control groups, respectively; *p* < 0.05). Patient satisfaction continued to improve until six months after TKA (+96 % and +88 % for treated and control groups, respectively; *p* < 0.001), but with no significant difference between groups (Fig. 5). It is noteworthy that one month after surgery, the SF-36 pain evaluation showed a significant improvement for the treated group only, thus confirming results of the VAS evaluation (*p* < 0.05) (data not shown).

### Long-term follow-up

**Fig. 6:**
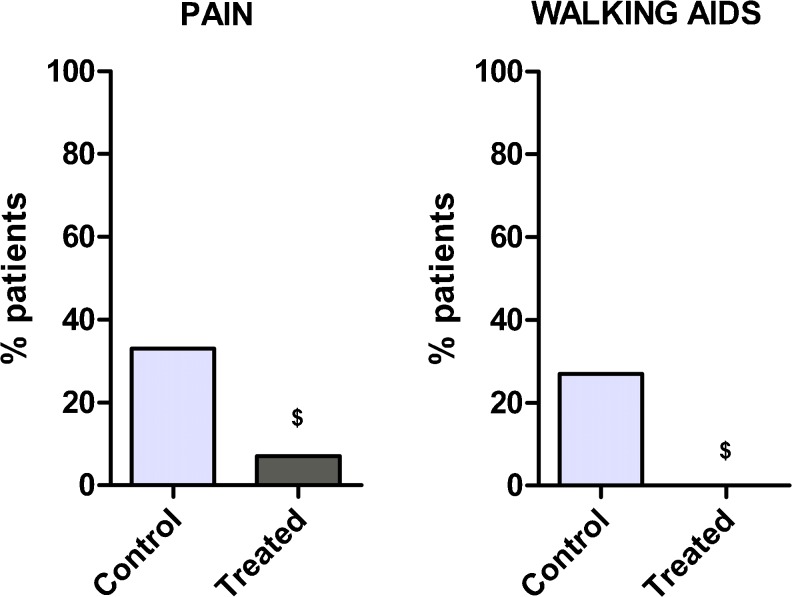
Long-term follow-up: percentage of patients still having severe pain (*left*) and occasionally requiring walking aids (*right*) 3 years after total knee arthroplasty (TKA). $*p* < 0.05 for the pulsed magnetic field (I-ONE) therapy group vs control group

Patients with persistent pain represented 7 % of the treated group and 33 % of the control group (*p* < 0.05). All the patients in the treated group reported walking without limitation or walking aids, whereas 27 % of patients in the control group occasionally used walking aids (*p* < 0.05) (Fig. 6).

## Discussion

Results of this study show that I-ONE therapy in patients who underwent TKA and the standard rehabilitation protocol significantly reduced pain and joint swelling and improved early functional recovery compared with patients who simply followed standard rehabilitation procedures. At one month follow-up, I-ONE therapy was particularly effective on knee swelling and on function, as standard rehabilitation alone did not allow significant improvement in these parameters with respect to pre-operative level. The earlier resolution of knee swelling observed using I-ONE therapy is remarkable, as it has been demonstrated to be highly important during the acute period after TKA to follow an adequate rehabilitation program [19]. Moreover, an inverse relationship between immediate functional recovery and inflammatory response after hip and knee arthroplasty is reported [8, 20]. These observations support the idea that the positive effect of I-ONE therapy on early outcome can be explained by a reduction in joint inflammation, as it has already been demonstrated that I-ONE therapy acts through the agonist activity for the A_2A_ adenosine receptors, leading to reduced local inflammatory processes [11–13, 21–23]. This is particularly relevant in patients undergoing TKA, as local joint inflammation originating from the severe osteoarthritis and from the surgical procedure [9] is often associated with abundant swelling and pain. Among the nonoperative measures useful to improve functional recovery of patients undergoing total joint replacement, PEMF treatment is scarcely investigated [23], and to our knowledge, only two clinical studies have been published [16, 24].

In our study, functional recovery and pain reduction were observed in both groups and improved until six months. Nevertheless, within the first two months after TKA, results were more evident among I-ONE therapy patients, with statistically significant differences with respect to untreated patients. One of the most important findings of this study is that control patients who reported pain two months after TKA also complained of pain at the three year follow-up. Moreover, patients in the treated group experienced faster resolution of pain and joint swelling, and only one patient reported pain requiring the use of NSAIDs at the three year follow-up. These observations again confirm that the early resolution of local inflammatory response after TKA is essential to enable patients to follow an adequate rehabilitation program attain functional recovery and especially to achieve better the long-term results [4, 19, 23]. As no signs of instability or incorrect component positioning were observed in either group after TKA, and no patient was scheduled for a second surgery on the same knee, our observations confirm that medium- to long-term results do not strictly depend on biomechanical outcome only but that important functional limitations can derive from an excessive reaction of joint soft tissues, including tissue fibrosis, heterotopic ossification and synovial reaction. These complications can be prevented by I-ONE therapy, which facilitates inflammation resolution and favours joint soft tissues healing by inhibiting the release of PGE_2_ and proinflammatory cytokines IL-6 and IL-8 and by stimulating the release of IL-10.

Interestingly, Feng et al. [25] show that pre-operative administration of cyclooxygenase-2 inhibitor can greatly ameliorate pain associated with TKA, reducing general and local inflammatory reactions. In light of this study, we could consider evaluating the efficacy of pre-operative I-ONE therapy on final TKA outcome.

The main limitation of this study is the lack of a double-blind condition, as a placebo group was not included. However, physicians who performed patient evaluations were blinded to the assigned group, and thus, the objective evaluations are reliable.

As the percentage of patients expected to experience pain or functional limitation after TKA is significantly higher than after total hip arthroplasty, the advantages deriving from early control of joint inflammation can certainly justify the use of I-ONE therapy in the first two months postoperatively and should be considered an effective completion of the surgical procedure.

## References

[1] Bourne RB, Chesworth BM, Davis AM, Mahomed NN, Charron KD (2010). Patient satisfaction after total knee arthroplasty: who is satisfied and who is not?. Clin Orthop Relat Res.

[2] Noble PC, Conditt MA, Cook KF, Mathis KB (2006). The John Insall Award: Patient expectations affect satisfaction with total knee arthroplasty. Clin Orthop Relat Res.

[3] Franklin PD, Li W, Ayers DC (2008). The Chitranjan Ranawat Award: functional outcome after total knee replacement varies with patient attributes. Clin Orthop Relat Res.

[4] Beswick AD, Wylde V, Gooberman-Hill R, Blom A, Dieppe P (2012). What proportion of patients report long-term pain after total hip or knee replacement for osteoarthritis? A systematic review of prospective studies in unselected patients. BMJ Open.

[5] Bonnin MP, Basiglini L, Archbold HA (2011). What are the factors of residual pain after uncomplicated TKA?. Knee Surg Sports Traumatol Arthrosc.

[6] Takahashi M, Miyamoto S, Nagano A (2002). Arthroscopic treatment of soft-tissue impingement under the patella after total knee arthroplasty. Arthroscopy.

[7] Sellam J, Berenbaum F (2010). The role of synovitis in pathophysiology and clinical symptoms of osteoarthritis. Nat Rev Rheumatol.

[8] Ugraş AA, Kural C, Kural A, Demirez F, Koldaş M, Cetinus E (2011). Which is more important after total knee arthroplasty: local inflammatory response or systemic inflammatory response?. Knee.

[9] Gandhi R, Santone D, Takahashi M, Dessouki O, Mahomed NN (2013). Inflammatory predictors of ongoing pain 2 years following knee replacement surgery. Knee.

[10] Tucci MA, Tsao AK, Lemos MB, Hughes JL (1997). Biochemical and immunochemical evaluation of tissues and synovial fluid from patients undergoing total joint arthroplasty. Biomed Sci Instrum.

[11] Varani K, De Mattei M, Vincenzi F, Gessi S, Merighi S, Pellati A, Ongaro A, Caruso A, Cadossi R, Borea PA (2008). Characterization of adenosine receptors in bovine chondrocytes and fibroblast-like synoviocytes exposed to low frequency low energy pulsed electromagnetic fields. Osteoarthritis Cartilage.

[12] Varani K, Vincenzi F, Tosi A, Targa M, Masieri FF, Ongaro A, De Mattei M, Massari L, Borea PA (2010). Expression and functional role of adenosine receptors in regulating inflammatory responses in human synoviocytes. Br J Pharmacol.

[13] Ongaro A, Varani K, Masieri FF, Pellati A, Massari L, Cadossi R, Vincenzi F, Borea PA, Fini M, Caruso A, De Mattei M (2012). Electromagnetic fields (EMFs) and adenosine receptors modulate prostaglandin E(2) and cytokine release in human osteoarthritic synovial fibroblasts. J Cell Physiol.

[14] Zorzi C, Dall’oca C, Cadossi R, Setti S (2007). Effects of pulsed electromagnetic fields on patients’ recovery after arthroscopic surgery: prospective, randomized and double-blind study. Knee Surg Sports Traumatol Arthrosc.

[15] Benazzo F, Zanon G, Pederzini L, Modonesi F, Cardile C, Falez F, Ciolli L, La Cava F, Giannini S, Buda R, Setti S, Caruso G, Massari L (2008). Effects of biophysical stimulation in patients undergoing arthroscopic reconstruction of anterior cruciate ligament: prospective, randomized and double blind study. Knee Surg Sports Traumatol Arthrosc.

[16] Moretti B, Notarnicola A, Moretti L, Setti S, De Terlizzi F, Pesce V, Patella V (2012). I-ONE therapy in patients undergoing total knee arthroplasty: a prospective, randomized and controlled study. BMC Musculoskelet Disord.

[17] Bankes MJ, Back DL, Cannon SR, Briggs TW (2003). The effect of component malalignment on the clinical and radiological outcome of the Kinemax total knee replacement. Knee.

[18] Soderberg GL, Ballantyne BT, Kestel LL (1996). Reliability of lower extremity girth measurements after anterior cruciate ligament reconstruction. Physiother Res Int.

[19] Mizner RL, Petterson SC, Clements KE, Zeni JA, Irrgang JJ, Snyder-Mackler L (2011). Measuring Functional Improvement After Total Knee Arthroplasty Requires Both Performance-Based and Patient-Report Assessments A Longitudinal Analysis of Outcomes. J Arthroplasty.

[20] Hall GM, Peerbhoy D, Shenkin A, Parker CJ, Salmon P (2001). Relationship of the functional recovery after hip arthroplasty to the neuroendocrine and inflammatory responses. Br J Anaesth.

[21] Ongaro A, Pellati A, Masieri FF, Caruso A, Setti S, Cadossi R, Biscione R, Massari L, Fini M, De Mattei M (2011). Chondroprotective effects of pulsed electromagnetic fields on human cartilage explants. Bioelectromagnetics.

[22] Aaron RK, Boyan BD, Ciombor DM, Schwartz Z, Simon BJ (2004). Stimulation of growth factor synthesis by electric and electromagnetic fields. Clin Orthop Relat Res.

[23] Ibrahim MS, Khan MA, Nizam I, Haddad FS (2013). Peri-operative interventions producing better functional outcomes and enhanced recovery following total hip and knee arthroplasty: an evidence-based review. BMC Med.

[24] Dallari D, Fini M, Giavaresi G, Del Piccolo N, Stagni C, Amendola L, Rani N, Gnudi S, Giardino R (2009). Effects of pulsed electromagnetic stimulation on patients undergoing hip revision prostheses: a randomized prospective double-blind study. Bioelectromagnetics.

[25] Feng Y, Ju H, Yang B, An H (2008). Effects of a selective cyclooxygenase-2 inhibitor on postoperative inflammatory reaction and pain after total knee replacement. J Pain.

